# Epidemiology of ADHD coming of age and a plea for prospective research on causes and consequences of ADHD throughout the lifespan in multidisciplinary team science

**DOI:** 10.1002/jcv2.12178

**Published:** 2023-06-01

**Authors:** Catharina A. Hartman

**Affiliations:** ^1^ Interdisciplinary Center of Psychopathology and Emotion Regulation (ICPE) University Medical Center Groningen (UMCG), University of Groningen Groningen The Netherlands

**Keywords:** ADHD, causality, comorbidity, developmental epidemiology, lifecourse, multidisciplinary research, prevention, risk and protective factors, team science

Attention deficit/hyperactivity disorder (ADHD) used to be studied at age of diagnosis–the typical time that professional help is sought and children get an ADHD diagnosis was also the time that they typically enrolled in scientific studies. As a consequence, most of our knowledge is about referred children with ADHD in middle childhood in the age range of 6–12 years. One implication of this is that for a long time ADHD research was not bothered so much by studying causes of ADHD *after* its onset—in other words, within the total of studies aimed at understanding the causes of ADHD, prospective studies in ADHD research have been rare.

The first paper that I will highlight in this editorial is by Miller et al. (2023) who discuss the development of ADHD in the period from conception to age of onset. They stress that for understanding the causes of ADHD prospective research prior to its onset is needed and explain that any differences observed in children with ADHD (i.e., after its onset) compared to children without ADHD may be secondary to personal and environmental alterations that are evoked by the ADHD symptoms themselves. At the same time, they point out that an onset of ADHD is gradual and the distinction between pre‐onset and post‐onset not clear‐cut: precursor behaviors of ADHD may already evoke personal and environmental alterations. Thus, for a causal understanding of ADHD, we need to know these as well. Prospective research from conception to full clinical onset allows us to chart such alterations and their temporal sequence up to a full onset of ADHD.

The value of establishing the temporal sequence of personal and environmental alterations leading to a clinical onset of a disease cannot be underestimated. It is one element (albeit by no means a sufficient element) in establishing the causality of a risk factor (the risk factor should occur prior to the onset of the disease), and a very important one in observational research. The accumulation of knowledge as to whether a risk factor is (likely) causal is necessary if we want to target the risk factor for interventions: that is, only causal risk factors can actually influence the outcome. Prospective research charting the sequences of personal and environmental alterations in risk during the gradual unfolding of ADHD over time provides opportunities to identify mediators that are useful as intervention targets in particular developmental periods. Also, prospective research may identify protective factors, by comparing children who have the same risk profile (e.g., at conception, at birth) but one group progressing to a full clinical onset of ADHD yet another not. That is, if we only study those who already have an onset of ADHD like we used to do we can never know if onset of ADHD (particularly the impairments experienced by the children) can be prevented, postponed or reduced in severity and thus if and how a developmental trajectory heading toward onset of ADHD can be shifted toward a more favorable outcome.

The paper by Miller et al. in this issue of JCPP Advances describes a research agenda for prospective research pre‐onset to ADHD. The authors have formed the Early ADHD Consortium to improve future prospective research on the causes of ADHD. An important factor herein in my view is sample size. Clearly, we need the statistical power coming from large samples to get robust insights into the causes of ADHD: ADHD is a multifactorial condition resulting from the collective influence of multiple personal and environmental risk factors, each contributing a minor part but working in unison to heighten ADHD susceptibility. With none of the risk factors being necessary or sufficient, pathways toward onset of ADHD diverge among children and the to‐be‐expected small effects of any risk factor manifest as even smaller effects when averaged across all children in the study sample. This reasoning on why large samples are needed holds all the more if we are to identify the interplay (interactions) among risk factors. Furthermore, Miller et al. point out that neurodevelopmental alterations likely emerge prior to overt behavioral changes and that we need to understand causal factors at additional levels of understanding (e.g., metabolic or neural levels) next to the overt behavioral level, emphasizing once more the need for large samples. Team science in consortia to enlarge the sample turned out to be the game changer in the field of genetics, and this should also work out to improve our understanding of the broader (potentially interacting) causes of ADHD. JCPP *Advances* is therefore proud to publish the “Delineating early developmental pathways to ADHD: Setting an international research agenda” from the Early ADHD Consortium.

The second paper that I will highlight by Li et al. is also published in JCPP Advances (2023) and focuses on a different phase in the life course of individuals with ADHD. Li et al. are a group of authors who come from another recently formed ADHD consortium “TIMESPAN,” which zooms in on cardiometabolic diseases which in most cases have an onset (long) after the onset of ADHD. The meta‐analysis by Li et al. is on cardiovascular diseases (CVD) and shows that adults with ADHD are nearly twice as likely to develop CVD than adults without ADHD. An important asset of the paper is that the authors embedded this finding in the broader available knowledge on the link between neuropsychiatric disorders and CVD, showing that the two‐fold risk in relation to CVD is similar to estimates for schizophrenia and substance use disorder but larger than estimates for mood, anxiety and stress related disorders. This group from TIMESPAN has just published a similar meta‐analysis on ADHD and type 2 diabetes (Garcia‐Argibay et al., 2023), and a strong empirical study on ADHD and CVD (Li et al., 2022), and together with the evidence from the longer‐standing research tradition on comorbidity of ADHD with obesity (Cortese et al., 2016; Nigg et al., 2016), we can conclude that the link between ADHD and cardiometabolic diseases has now been well‐established. Together, these papers including the one by Li et al. in JCPP Advances discuss steps that need to be taken for a mechanistic understanding of the causes of an onset of comorbid cardiometabolic diseases in adults with ADHD as well as the consequences (particularly the consequences are studied in the TIMESPAN consortium). Like causes of ADHD need to be established pre‐onset to ADHD, prospective research on the sequence of personal and environmental alterations leading up to a comorbid condition need to be established pre‐onset of a comorbid condition. This, with the same complication as in ADHD that onset of a comorbid condition is gradual and does not only include overt alterations in personal risk factors but also, for example, metabolic or neural alterations. One important reason to form the TIMESPAN consortium was, like it likely was for the Early ADHD Consortium, the need for a large sample size to do prospective research on multifactorial conditions (in this case both ADHD and the different cardiometabolic diseases are multifactorial).

As argued, the ideas put forward by Miller et al. are not only relevant pre‐onset to ADHD but throughout the lifespan. To illustrate this further, I want to highlight two aspects specifically. First, as pointed out already, Miller et al. emphasize that environmental risk factors of ADHD in early development may change over time. As an example of altering risk factors over development, maternal immune activation, during pregnancy, may contribute to the risk of a later onset of ADHD (He et al., 2022), while parental difficulties to regulate their emotions, subsequently during toddlerhood, may at that point in time contribute to the risk of a later onset of ADHD (Claussen et al., 2022). Note that these findings, apart from that they need further study, have never been studied prospectively in the same children to establish potential accumulation of risk. We may extend the idea that risk factors change across the lifespan from the causes of ADHD to the course of ADHD. For example, with regard to the development of comorbid obesity, impulsive reward driven eating of high caloric, low nutrient, foods leading to overweight may be particularly prevalent in adolescents with ADHD, while lack of physical activity associated with difficulties with motivation and organization in ADHD may contribute to weight gain particularly in adults (note that this is a fictitious example, changes in risk factors contributing to weight gain in individuals with ADHD relative to individuals without ADHD in different developmental periods have not yet been studied). These examples on changing risk factors across the lifespan illustrate how much prospective work, both pre‐onset to or post‐onset of ADHD, is still to be done.

A second example of how ideas put forward by Miller et al. on causes of ADHD can be extended across the lifespan relates to the specificity or non‐specificity of personal and environmental risk factors of ADHD (non‐specificity meaning that they also play role in the onset of other conditions and not just in the onset of ADHD). Miller et al. explain that how an exclusive focus on onset of ADHD and the presumption of specificity of risk for this outcome may obscure transdiagnostic patterns and lead to inaccurate causal models. That non‐specificity is ubiquitous and also relevant in the course of ADHD is undisputed. I already cited the example reported by Li et al. on the association of ADHD with CVD amidst similar associations of schizophrenia, substance use disorder, mood, anxiety and stress related disorders with CVD. Similarly, the most recent Genomewide Association Study on ADHD showed high sharing of genetic (concordant and discordant) genetic variants of ADHD with other disorders showing even more than before their strong intertwinement of the genetic architecture (Demontis et al., 2023). Specifically with regard to the onset of comorbid conditions during the course of ADHD, we may first of all draw from known personal and environmental risks of these conditions. The pertinent question with regard to specificity in this context is if these known risk factors have a similar effect whether ADHD is present or not (i.e., an interaction effect). For example, the effect may be stronger in individuals with ADHD compared to individuals without ADHD as in the previous example on how weight gain may be specifically linked to ADHD characteristics in different developmental periods (impulsivity and reward drivenness; motivational and organizational difficulties). In addition, prospective research may potentially show that timing may be different: known risk factors and precursors of onset of comorbidity may exert their influence earlier in individuals with than without ADHD. For example, there is some evidence that the onset of a condition is earlier in persons with than without ADHD (e.g., substance abuse: Dunne et al., 2014; CVD, Li et al., 2022). In addition, there may be risk factors that are specific to individuals with ADHD that lead up to the comorbid condition and which we currently do not know. Close monitoring in prospective research, as advocated by Miller et al., but now applied to the unfolding of the course of ADHD, specifically onset of comorbid conditions, will help establish this knowledge. This second example on the specificity of personal and environmental risk factors shows that broad knowledge beyond just ADHD is required. For example, in the example of Timespan, both specialists on ADHD and on cardiometabolic diseases take part. Again, if extended to other levels of understanding (metabolomic, neural, etc.), in addition to the overt personal risk factors, this holds even more. Thus, not only large sample sizes but also broad knowledge to do the best possible research can be accomplished by multidisciplinary team science.

In this editorial, I argued that making the distinctions between the causes of ADHD and the course of ADHD and between the causes of onset of comorbidity and the course thereafter are essential. At the same time, the gradual onset of ADHD and any comorbid condition makes clear that there is no hard cut between causes and consequences. Yet through close monitoring over time, we will be able to establish the cascades of personal and environmental alterations leading to the onset of ADHD and to the onset of comorbid conditions. In my ideal world, we would do prospective mechanistic research on ADHD (or any other condition) from pregnancy to death, but this is obviously not going to happen. Yet, papers from multidisciplinary research teams working together on different parts of the lifespan, like Miller et al. in the Early ADHD Consortium, and Li et al. in the Timespan consortium, show that we are making good progress in understanding the ADHD lifespan.

## AUTHOR CONTRIBUTIONS

**Catharina A. Hartman**: Conceptualization; writing–original draft; writing–review and editing.

## CONFLICT OF INTEREST STATEMENT

Catharina A. Hartman *is part of the TIMESPAN consortium, but not a co‐author on the cited papers.* She is also Deputy Editor‐in‐Chief for JCPP *Advances*.
